# RETRACTION: 27‐Gauge Microincision Vitrectomy Surgery Compared With 25‐Gauge Microincision Vitrectomy Surgery on Wound Closure and Need for Wound Suture and Other Postoperative Parameters in the Treatment of Vitreoretinal Disease: A Meta‐Analysis

**DOI:** 10.1111/iwj.70463

**Published:** 2025-04-02

## Abstract

**RETRACTION:** LiS. , LiY. , WeiL. , FangF. , JiangY. , ChenK. , YangX. , and LiuH. , “27‐Gauge Microincision Vitrectomy Surgery Compared With 25‐Gauge Microincision Vitrectomy Surgery on Wound Closure and Need for Wound Suture and Other Postoperative Parameters in the Treatment of Vitreoretinal Disease: A Meta‐Analysis,” International Wound Journal 20, no. 3 (2023): 740–750, 10.1111/iwj.13917.36787269 PMC9927918

The above article, published online on 04 August 2022, in Wiley Online Library (http://onlinelibrary.wiley.com/), has been retracted by agreement between the journal Editor in Chief, Professor Keith Harding; and John Wiley & Sons Ltd. Following an investigation by the publisher, all parties have concluded that this article was accepted solely on the basis of a compromised peer review process. The editors have therefore decided to retract the article. The authors did not respond to our notice regarding the retraction.

**RETRACTION:** S. Li , Y. Li , L. Wei , F. Fang , Y. Jiang , K. Chen , X. Yang , and H. Liu , “27‐Gauge Microincision Vitrectomy Surgery Compared With 25‐Gauge Microincision Vitrectomy Surgery on Wound Closure and Need for Wound Suture and Other Postoperative Parameters in the Treatment of Vitreoretinal Disease: A Meta‐Analysis,” International Wound Journal 20, no. 3 (2023): 740–750, 10.1111/iwj.13917.36787269 PMC9927918

The above article, published online on 04 August 2022, in Wiley Online Library (http://onlinelibrary.wiley.com/), has been retracted by agreement between the journal Editor in Chief, Professor Keith Harding; and John Wiley & Sons Ltd. Following an investigation by the publisher, all parties have concluded that this article was accepted solely on the basis of a compromised peer review process. The editors have therefore decided to retract the article. The authors did not respond to our notice regarding the retraction.

